# Why does viral RNA sometimes persist after recovery from acute infections?

**DOI:** 10.1371/journal.pbio.3001687

**Published:** 2022-06-01

**Authors:** Diane E. Griffin

**Affiliations:** W. Harry Feinstone Department of Molecular Microbiology and Immunology, Johns Hopkins Bloomberg School of Public Health, Baltimore, Maryland, United States of America

## Abstract

DNA viruses often persist in the body of their host, becoming latent and recurring many months or years later. By contrast, most RNA viruses cause acute infections that are cleared from the host as they lack the mechanisms to persist. However, it is becoming clear that viral RNA can persist after clinical recovery and elimination of detectable infectious virus. This persistence can either be asymptomatic or associated with late progressive disease or nonspecific lingering symptoms, such as may be the case following infection with Ebola or Severe Acute Respiratory Syndrome Coronavirus 2 (SARS-CoV-2). Why does viral RNA sometimes persist after recovery from an acute infection? Where does the RNA come from? And what are the consequences?

## Abstract 2

Most RNA viruses cause acute infections that are cleared from the host as they lack the mechanisms to persist; however, phenomena such as "long COVID" suggest that viral RNA can persist after clinical recovery and elimination of detectable infectious virus. This Unsolved Mystery article explores the meaning, origins and consequences of such persistent RNA.

## Introduction

**Fig 1:**
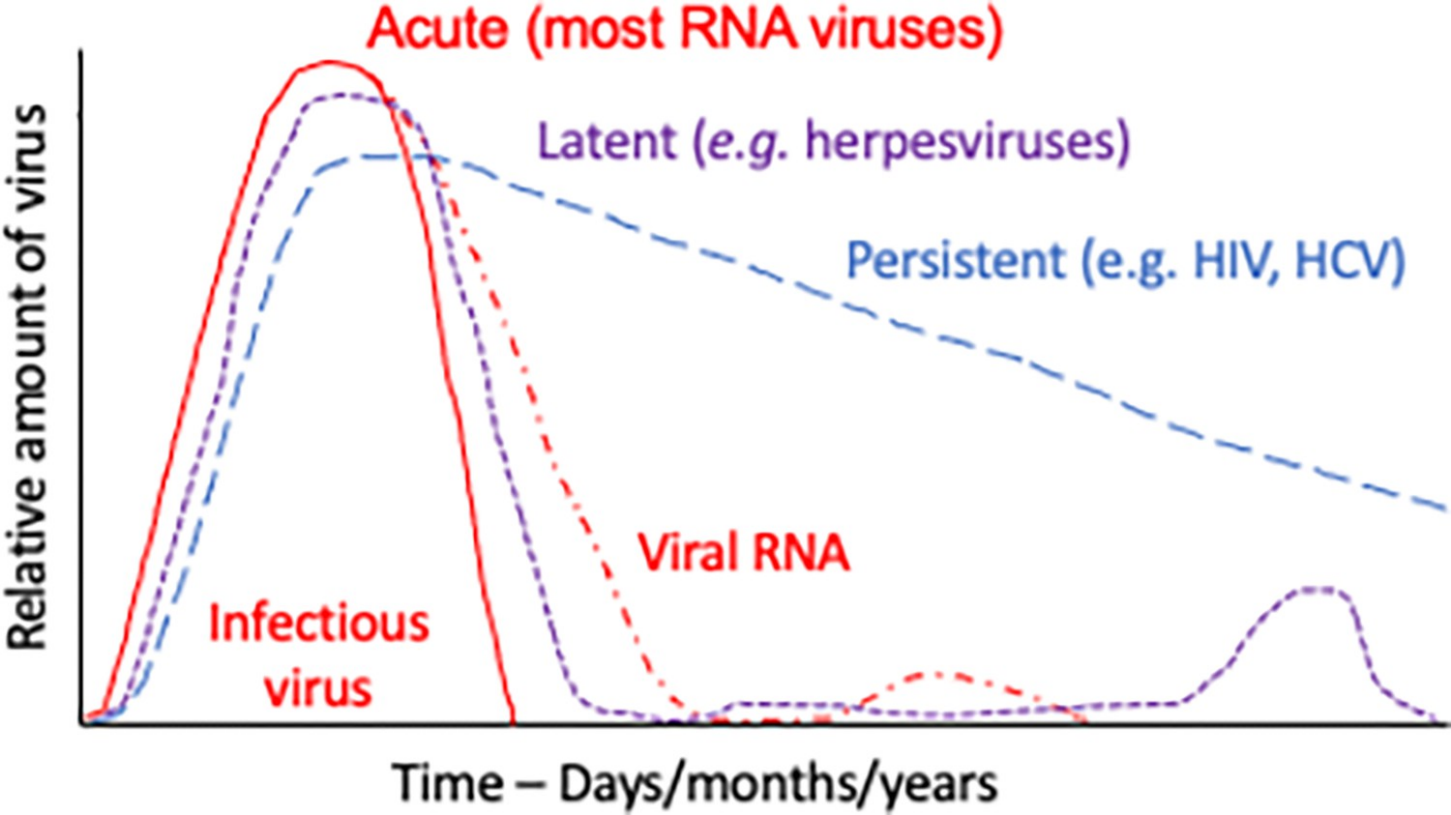
Patterns of virus production over time that maintain human viruses within the population. Representative patterns are shown for RNA viruses often associated with persistent RNA that can cause late complications and occasionally reactivate (red), viruses that establish latency and reactivate (such as herpesviruses) (purple), and viruses not cleared by the immune response that continue to produce infectious virus (such as HIV and HCV) (blue). HCV, hepatitis C virus; HIV, human immunodeficiency virus.

Viruses are obligate intracellular infectious agents that are maintained in a population by continuous transmission to new susceptible individuals. In the absence of a reservoir, such as an insect vector or animal population capable of facilitating transmission to humans, viruses require alternative strategies to remain within human populations (Fig 1). Herpesviruses (such as varicella, herpes simplex, or Epstein–Barr) are DNA viruses with an optimum strategy, because after the acute infection resolves and production of infectious virions ceases, they become latent and can reactivate (in the form of shingles, mucosal ulcers, or asymptomatic shedding) to produce infectious virions months, years or decades later to infect a new group of susceptible people [1–3]. Of the RNA viruses, some (such as hepatitis C virus (HCV) and human immunodeficiency virus (HIV)) can evade immune control and continuously produce infectious virions [4–6]. Because these viruses do not cause rapidly lethal disease and can be transmitted over a long period of time, transmission does not need to be efficient. However, most acute viral infections are caused by RNA viruses that produce disease for a relatively short period of time and are associated with recovery and immunity to reinfection (e.g., measles, rubella, polio, and hepatitis A viruses) [7]. For these acute RNA viral infections, infectious virions are produced only transiently, so transmission to new susceptible hosts during this time must be efficient. Because these viruses must find and infect susceptible people in the population during the acute phase of disease to avoid dying out, they may become targets for eradication [8].

However, it has become increasingly clear that recovery, elimination of infectious virus, and development of immunity to acute nonretroviral RNA viruses do not necessarily mean simultaneous elimination of the viral RNA [9–22]. The need to understand the pathophysiology of the prolonged symptoms that for many complicate recovery after infection with Severe Acute Respiratory Syndrome Coronavirus 2 (SARS-CoV-2)—so-called “long Coronavirus Disease (COVID)” or post-acute sequelae of COVID-19 (PASC)—has recently called attention to the potential role of RNA persistence in causing specific late complications, as well as in preventing complete recovery from acute infection [23–28]; consequences are also seen following other acute RNA virus infections (Table 1). But how and why does viral RNA persist, often without evidence of infectious virus, and what are the potential consequences of this persistence for human disease? These questions will form the basis of discussions in this Unsolved Mystery.

**Table 1:** Potential sites and consequences of RNA persistence after human infection with acute nonretroviral RNA viruses.

Virus	Sites of RNA persistence	Cell type	Consequences	References
***Picornavirus***				
Rhinovirus	Respiratory tract	Epithelial cells?	Asthma	[29]
Enterovirus	Heart	Cardiac myocytes	Cardiomyopathy	[30]
Hepatitis A	Liver	Hepatocytes	Late hepatitis relapse	[7,15]
Polio	Brain and spinal cord	Motor neurons	Late progression of paralysis and fatigue	[19,31]
***Alphavirus***				
Chikungunya	Joints	Macrophages	Persistent joint pain	[10]
Ross River	Joints	Macrophages	Persistent joint pain	[32]
Sindbis	Joints	Macrophages?	Persistent joint pain	[33]
***Flavivirus***				
Zika	Testes	Sertoli cells	Late sexual transmission	[11,34]
Japanese encephalitis	Brain	Neurons	Encephalitis relapse and Parkinson-like disease	[35]
West Nile	Kidney?	Unknown	Kidney failure?	[36]
Tick-borne encephalitis	Brain	Neurons	Late progressive encephalitis	[37]
***Coronavirus***				
SARS-CoV-2	Respiratory tract and intestine	Epithelial cells and macrophages?	Long COVID/PASC?	[18,38]
***Arenavirus***				
Lassa	Testes, kidney, and respiratory tract	Sertoli cells?	Epididymitis	[39,40]
***Paramyxovirus***				
Measles	Lymphoid tissue and brain	Lymphocytes, monocytes, and neurons	Life-long immunity; late progressive CNS disease (SSPE)	[41]
Respiratory syncytial	Respiratory tract	Epithelial cells and macrophages?	Chronic pulmonary disease	[42–44]
***Filovirus***				
Ebola	Testes, eye, and brain	Endothelial cells and macrophages	Late sexual transmission; recurrent/progressive uveitis and encephalitis; postviral syndrome	[45–49]
Marburg	Testes	Sertoli cells	Late sexual transmission	[50]

CNS, central nervous system; COVID, Coronavirus Disease; PASC, post-acute sequelae of COVID-19; SARS-CoV-2, Severe Acute Respiratory Syndrome Coronavirus 2; SSPE, subacute sclerosing panencephalitis.

## Where does viral RNA persist?

The occurrence of long-term persistence of viral RNA has been known for decades, particularly in sites with specialized relationships to the immune system (so-called “immune-privileged” sites such as the brain, eyes, and testes), an early example being the identification of measles virus as the cause of subacute sclerosing panencephalitis (SSPE), a progressive fatal central nervous system (CNS) disease that becomes manifest many years after apparent recovery from the original acute measles virus infection [51–53]. More recently, late appearance of uveitis (Box 1) and recurrence of encephalomyelitis (Box 1) due to Ebola virus infection have emphasized the importance of RNA persistence in the eye, as well as the brain, and the potential for causing progressive disease [47 49]. Sexual transmission of Zika, Marburg, and Ebola viruses months to years after recovery from acute disease has also highlighted the importance of virus persistence in the testes for triggering new chains of transmission and transfer to new geographic regions [34,54–57].

**Box 1. Definition of key terms used in this article**

- Adaptive immune response—production of virus-specific antibodies and T cells
- Antigen—viral component, usually a protein, which stimulates production of virus-specific antibodies and T cells
- Cardiomyopathy—dysfunction of the heart muscle
- Cytolytic—causing death of a cell due to lysis
- CpG—pairing of cytosine and guanosine in nucleic acid that is unusual in cellular RNA and DNA
- Encephalomyelitis—inflammation of the brain and spinal cord that can be a response to viral infection
- Immunocytochemical assays—methods for microscopically visualizing proteins, such as viral proteins, in cells using antibody to the protein
- Innate immune mechanisms—intrinsic cellular responses to infection that usually occur rapidly and can often control pathogen replication and spread prior to induction of adaptive immune responses
- MHC class I—polymorphic MHC; molecule that can bind viral peptides produced by infected cells, displaying them on the cell surface for presentation to virus-specific CD8 T cells that may be able to kill the infected cell
- Peripheral blood mononuclear cells—lymphocytes and monocytes present in circulating blood that come primarily from bone marrow and lymphoid tissue and may infiltrate sites of infection
- Ribonucleocapsid—viral RNA surrounded by nucleocapsid protein
- Reverse transcriptase polymerase chain reaction (RT-PCR)—it is a method for converting RNA into a DNA copy for subsequent amplification using a thermostable DNA polymerase and primers specific for the gene of interest. The amplified product can be quantified or sequenced.
- Uveitis—inflammation of the uvea, which is the middle vascular layer of the eye

**Fig 2:**
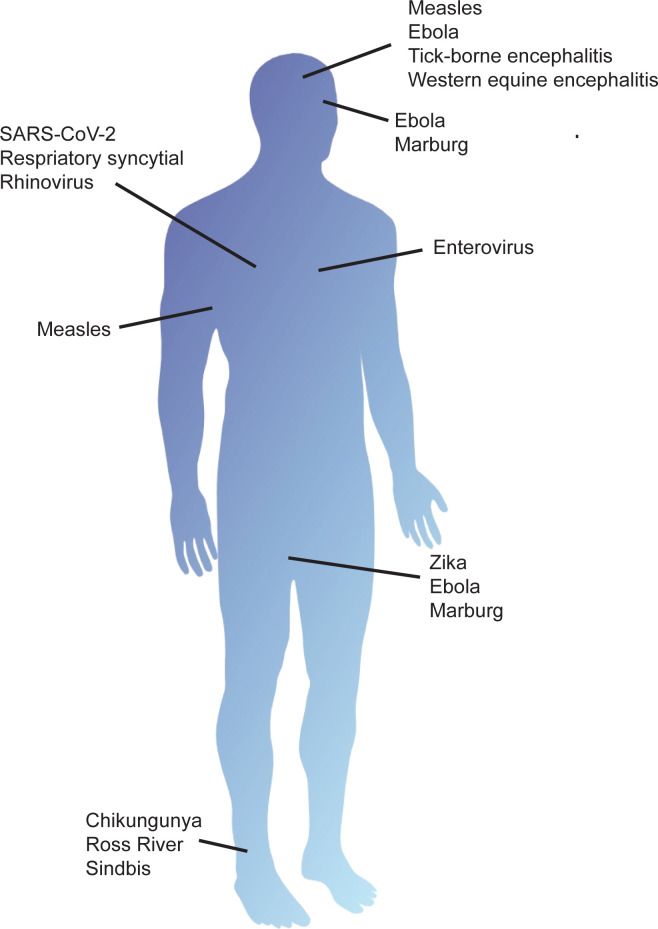
Sites of RNA persistence following infection. Tissues in which RNA viruses persist after infection include the nervous system, eyes, joints, lymph nodes, heart, respiratory tract, and testes. SARS-CoV-2, Severe Acute Respiratory Syndrome Coronavirus 2.

However, viral RNA persistence is not restricted to sites classically considered immune privileged, but can also occur in other sites including blood, lymphoid tissue, joints, respiratory tract, gastrointestinal tissues, and kidney, with a variety of known and unknown consequences [12,13,14,58–61] (Table 1, Fig 2). Organ-specific problems include chronic joint pain after infection with alphaviruses such as chikungunya, Ross River, and Sindbis that acutely cause rash and arthritis [10,32,62], cardiomyopathy (Box 1) after enterovirus infection [30], asymptomatic shedding of respiratory viruses [63], and chronic pulmonary disease associated with respiratory syncytial virus (RSV) and rhinovirus persistence [29,42,43]. Consequences may also include more nonspecific postviral syndromes such as PASC, post-Ebola, and post-polio syndromes, characterized by symptoms including fatigue, headache, muscle pain, and joint pain [23,31,64].

Viral RNA persistence in the absence of culturable virus is typically detected in RNA extracted from secretions, blood, or tissue samples. For long-lived cells such as neurons or cardiac myocytes, this RNA is presumed to come from the originally infected surviving cells present in those samples. However, few studies have attempted to identify or characterize the cellular source of the RNA detected, and clearance from some tissues may be more effective than from others. For example, after recovery of experimentally infected nonhuman primates from acute Ebola and Marburg filovirus infections, viral RNA is no longer detectable in primary sites of replication such as the liver but can often be found in the eyes and testes, where macrophages and Sertoli cells, respectively, remain RNA positive [45,46,50]. Tissue macrophages are also the sites of alphavirus RNA persistence in joints and Zika virus persistence in lymphoid tissues [10,12,65]. Prolonged detection of viral RNA in respiratory secretions, stool, sweat, conjunctival fluid, and urine likely comes from infected epithelial cells and is common even though these cells are relatively short lived and continuously replaced [11,18,36,58,61,66–68]. In measles virus infections, epithelial cells in multiple tissues, lymphocytes and monocytes in blood, and lymphoid tissue are prominent sites of infection [69,70]. Infectious virus is cleared during induction of the adaptive immune response and can no longer be cultured from any site shortly after resolution of the rash. However, viral RNA remains detectable in peripheral blood mononuclear cells (Box 1), respiratory secretions, and urine for weeks to months, and even longer in lymphoid tissue [14,41,61,68,71]. Little is known about the nature of the viral RNA that is detected in measles or other acute RNA viral infections or whether cells with viral RNA are the originally infected cells that survived acute infection and avoided immune elimination or newly infected cells through continued cell-to-cell transfer of viral RNA.

Detection of infectious virus is inherently less sensitive than detection of viral RNA and may be influenced by the presence of neutralizing antibody in the sample. Cocultivation of cells from tissues or secretions with susceptible cells is required to recover viruses such as measles but may not have been attempted for studies reporting the presence of viral RNA. Therefore, lack of detection of infectious virus may be due in part to differences in sensitivity and availability of the assays used. Development of techniques that can more easily identify the presence of assembled virions capable of initiating infection would provide increased understanding of the clearance and persistence of RNA viruses.

## What form of viral RNA persists in the absence of infectious virus?

Because infectious virus cannot be recovered and RNA is susceptible to degradation, it is often assumed that what is detected by reverse transcriptase polymerase chain reaction (RT-PCR; Box 1) is fragmented or degraded viral RNA [25]. However, several studies have shown the long-term presence of full-length RNA capable of resuming productive replication if immune control is relaxed [16,21,72–74]. Unexpected late transmission of Ebola, Marburg and Zika viruses attest to the presence of persistent full-length genomic RNA after apparent resolution of these infections [57,75–77].

For picornaviruses, positive-strand RNAs are detectable for longer than negative-strand RNAs, and for coronaviruses, genomic RNAs are detectable for longer than the subgenomic RNAs that are produced during active virus replication [78,79]. However, these differences may reflect the relative abundance of these RNAs, and for alphaviruses, subgenomic RNA, which is more abundant than genomic RNA, is often detectable for longer.

For Borna disease virus that replicates in the nucleus, persistently infected cells retain genomic RNA in aggregates of viral ribonucleoproteins tethered to host chromosomes with host nuclear proteins that are maintained in daughter cells through the cell cycle [80,81]. However, most RNA viruses replicate in the cytoplasm, and, therefore, this is the likely site for RNA to persist, although reverse transcription by cellular enzymes has been postulated as a mechanism of persistence for nonretroviral RNA viruses as endogenous viral elements [82,83]. In the cytoplasm, ribonucleocapsid structures (Box 1) may protect the RNA of negative-strand viruses, while association with membranous structures may protect the RNA of positive-strand viruses, but this hypothesis requires further investigation.

## How do RNA viruses evade the immune system to persist?

Innate immune mechanisms (Box 1) can control intracellular virus replication and target viral RNA for degradation, but adaptive immune responses are required for complete clearance of infected cells. Many intrinsic cellular antiviral mechanisms detect features of viral RNAs that are distinct from cellular RNAs, such as CpG content (Box 1), 5′ triphosphate, cap structure, and double-stranded RNA [84,85]. Recognition by innate sensors can target viral RNA for degradation or cause the inhibition of translation and replication and can activate pathways that result in the production of the signaling molecule interferon (IFN). Synthesis of IFN-stimulated antiviral proteins can further decrease virus replication and RNA synthesis [86]. Therefore, viral pathogens have often evolved RNA sequences and structures that circumvent induction of innate immune responses to promote virus replication and intracellular survival. However, adaptive immune responses consisting of virus-specific antibody and T cells are still induced.

**Fig 3:**
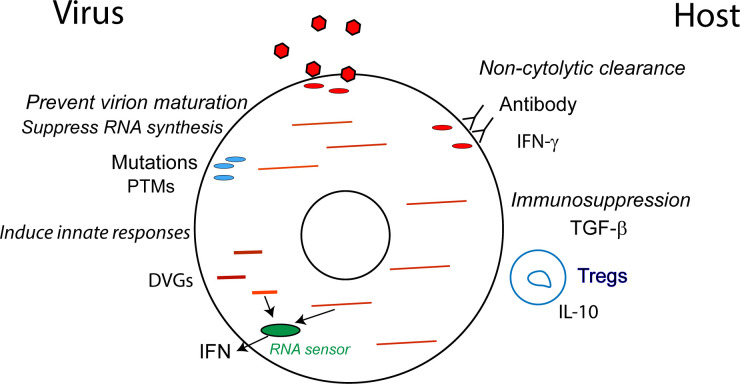
Mechanisms for suppressing production of infectious virions. Several mechanisms exist whereby the virus and host can suppress the production of infectious virions to facilitate the survival of infected cells and viral RNA persistence. For example, the virus may acquire mutations that decrease virion assembly, induce innate responses, or decrease RNA synthesis, while the host employs antiviral immune responses that facilitate infected cell survival. DVG, defective viral genome; IFN, interferon; IL, interleukin; PTM, posttranslational modification; TGF, transforming growth factor.

Complete clearance of virus and virus-infected cells requires both prevention of virus spread to new cells and elimination of previously infected cells, either through virus-induced or immune-mediated cell death. Although viruses frequently lyse cells in tissue culture, primary cells and cells infected in vivo are often resistant to induction of cell death. These cells activate intrinsic cellular pathways that promote survival and combine with both host and viral strategies to downregulate replication and prevent lethal damage to the infected cell [87] (Fig 3). Persistence can evolve in the infected host through rapid mutation and selection of less lytic viral variants. This evolutionary process is facilitated by the error prone RNA-directed RNA polymerases that characterize RNA viruses [88,89] and by editing enzymes in the host cell [90,91]. In addition, early treatment with antibody may promote persistent infection [92,93].

Immune mechanisms for eliminating virus-infected cells that survive infection include cell killing by cytotoxic cells such as natural killer cells, which recognize a lack of major histocompatibility complex (MHC) class I expression (Box 1), and CD8^+^ T cells that recognize viral peptides expressed in the context of MHC class I molecules. In addition, binding of antibodies to the infected cell surface can direct cells toward antibody-mediated cytotoxicity or phagocytosis by immune cells [94–96]. Therefore, immune-mediated clearance requires recognition of the infected cell by immune effector cells, primarily through changes in surface expression of host or viral proteins. However, adaptive immune-mediated virus clearance is not always cytolytic (Box 1). For essential cells that are not easily replaced, such as neurons, noncytolytic control is advantageous for the host [97,98]. Antibodies that recognize alphavirus surface glycoproteins are required for clearance of infectious virions from the brains of infected mice and act by inducing antiviral signaling cascades that suppress production of viral RNA and infectious virions and inhibit virus release without harming the infected neurons [96,99–103]. Thus, the infected neuron survives with viral RNA still present. T cells can also employ noncytolytic mechanisms for cell type–specific clearance of infectious virus through local production of cytokines with antiviral activity such as IFN-γ [104–107]. T cell cytotoxicity may also be actively suppressed, particularly in immune-privileged sites, by expression of suppressive cytokines (e.g., TGF-β) and preferential recruitment of regulatory T cells [50,108]. Thus, the adaptive immune response can employ several noncytolytic mechanisms for clearance of infectious virus that allow survival of cells that still harbor viral RNA (Fig 3).

### Strategies that avoid immune-mediated clearance of infected cells

To escape clearance, viruses must avoid both elimination by the immune response and killing of all infected cells, processes that are more likely to occur in some types of cells and tissues than in others. Avoiding immune-mediated clearance mechanisms requires the infected cell to become invisible to the immune system or unresponsive to cytolytic immune effectors by eliminating both surface expression of viral proteins and MHC presentation of viral peptides. Viruses infecting long-lived cells in immune-privileged tissues may be particularly likely to survive and retain persistent RNA after infection [11,19,21,50,109–113]. Several early studies of progressive tick-borne and western equine viral encephalitis conducted prior to the availability of sensitive methods for detecting viral RNA provided clinical and pathological evidence of RNA persistence and ongoing inflammation in the absence of infectious virus in the CNS [17,20,114–116]. As neurons (and likely other long-lived cells such as cardiac myocytes) mature and become fully differentiated, they acquire the ability to restrict virus replication and survive the stress of infection [117–119]. The mechanism(s) underlying differentiation-dependent susceptibility to virus infection have not been fully elucidated but likely involve both increased expression of innate factors that restrict virus replication and/or promote cell survival and decreased availability of factors required for virus replication in terminally differentiated cells [117,120].

Survival of infected cells is often accompanied by acquisition of viral mutations that foster persistence. For example, for viruses that are assembled and released from the cell surface, mutations that limit or prevent cell surface expression of viral proteins can prevent recognition by antibodies. In the measles virus-induced late disease SSPE, virion proteins required for particle assembly at the plasma membrane (hemagglutinin, fusion, and matrix) have acquired changes that prevent cell surface expression and virion assembly but promote cell-to-cell ribonucleoprotein transfer to uninfected cells, thereby allowing continued spread of viral RNA without producing infectious virions [121–124]. Similar mutations have been observed in the viral RNAs from persistent CNS infections due to mumps and mouse hepatitis viruses [113,125].

Persistence in cells that are replaced more frequently (e.g., endothelial cells, epithelial cells, lymphocytes, and monocytes) may continue for shorter periods of time. In lymphocytic choriomeningitis virus (LCMV) infection of cell fate reporter mice, noncytolytic clearance from hepatocytes is accompanied by continuous infection of new cells to maintain persistence [126]. Epithelial cells in the respiratory tract and elsewhere commonly permit rapid cell-to-cell transfer of viral nucleocapsids without release of virus from the cell surface that may foster persistence of detectable viral RNA long after infectious virions can be recovered [127]. It is not clear whether the observed slow decrease in levels of detectable viral RNA in peripheral blood mononuclear cells, urine, stool, and respiratory secretions (Fig 1) is due to turnover of these cells, RNA degradation, or eventual immune-mediated elimination [14].

### Strategies that avoid killing of infected cells

Avoiding virus-induced cell death usually requires limiting virus replication [87,128]. A variety of mechanisms are employed by viruses to restrict replication. For example, several RNA viruses (e.g., Borna disease virus, LCMV, coxsackievirus, and hantavirus) undergo 5′-terminal trimming of the genome that both suppresses replication and prevents the activation of innate immune responses [129–132]. Ebola virus genomes from the eyes of infected humans and ferrets have acquired stop codons in the polymerase gene that would limit RNA synthesis [133,134], and phosphorylation of the paramyxovirus P protein represses viral replication late in infection and fosters persistence [135].

Replication may also be restricted through activation of IFN pathways and expression of IFN-stimulated genes encoding antiviral proteins. For example, production of defective viral genomes (DVGs), particularly so-called “copy-back” DVGs, by many RNA viruses leads to induction of innate immune responses that control virus replication and permit persistence [136]. Copy-back DVGs are generated when the viral polymerase becomes detached from the template genome and switches to another genome template to duplicate the terminal end. These shorter incomplete genomes have a replicative advantage over full-length genomes and can induce both IFN and pro-survival pathways to promote persistence [137,138]. For example, in lung infection with RSV, early production of DVGs activates RIG-I-like receptors to stimulate the activation of IRF3 and IRF1, leading to production of TNFα, IFNλ, and IFIT1, suppression of virus replication, and survival of persistently infected cells [136,139,140]. DVGs have been demonstrated in the testes during filovirus infection of nonhuman primates [141] and in the lungs of children with RSV infection [140].

## What are the consequences of RNA persistence?

Viral RNA alone may stimulate innate immune responses and inflammation associated with IFN production to drive chronic inflammation [60]. However, viral RNA persistence without production of infectious virions is frequently accompanied by evidence of viral protein synthesis and T cell activation, indicating that viral RNA is being translated, if not replicated or assembled into culturable virus particles [10,142]. Viral protein can sometimes be detected by immunocytochemical assays [10,15,19,143] (Box 1), but such techniques are relatively insensitive compared with those for detecting RNA, and most often the evidence comes from ongoing or renewed stimulation of a local or systemic adaptive immune response [144]. For example, in mice that have recovered from acute rhabdovirus and influenza virus infections, passively transferred immune cells detect and are activated by persistent viral antigens [145,146] (Box 1). Although antigens may persist without ongoing translation of viral RNAs, longitudinal studies of measles and Ebola have identified recurrent waves of immune activation consistent with periodic increases in immune stimulation by viral proteins [71,147,148].

Consequences of chronic immune stimulation associated with persistent RNA are dependent on the site of persistence. For example, persistence of RNA in the CNS of mice that have recovered from acute alphavirus-induced encephalomyelitis is accompanied by detection of viral protein weeks after infection, and maintenance of B cells secreting antiviral antibodies and T cells producing IFN-γ for more than a year [100,149–152]. Likewise, oligodendrocytes surviving acute coronavirus infection with persistent RNA promote prolonged T cell residence and inflammation in the CNS [111,153]. This type of late CNS pathology may or may not be associated with progressive neurologic disease [17,115,154]. Persistence of alphavirus RNA in synovial tissues is linked to the prolonged inflammation and joint pain that many patients have after infection, and persistence of enteroviral RNA in the myocardium is associated with progressive cardiac dysfunction [10,30].

Determining the importance of RNA persistence is of particular relevance for understanding the failure to fully recover from acute infections such as occurs after SARS-CoV-2 infection and Ebola virus disease. PASC afflicts 30% to 50% of those recovering from COVID-19 [23] and encompasses a variety of symptoms that affect different organ systems including fatigue, brain fog, muscle weakness, gastrointestinal distress, cough, and shortness of breath [26,155]. Infectious virions in blood (viremia) have not been documented, but viral RNA in blood (RNAemia) is found in those with more severe disease, suggesting systemic spread of infection, and is predictive of PASC [27,28]. Those with persistent symptoms at 3 months after acute disease are more likely to have increased levels of pro-inflammatory cytokines (e.g., TNF) and chemokines (e.g., IP-10 and MCP-1), as well as factors associated with vascular injury (e.g., VCAM-1 and ICAM-1) [156]. Prolongation of symptoms due to ongoing immune stimulation is suggested by identification of viral RNA and protein in a subset of monocytes [143]. The importance of persistent viral RNA relative to inflammation, autoimmunity, or reactivation of latent infection with other viruses (e.g., Epstein–Barr virus) in the pathogenesis of PASC remains to be determined, but PASC is likely to be more than one disease with multiple contributing factors [28]. Persistent RNA could continue to stimulate innate immune responses, but protein translation would be needed for continued activation of adaptive immune responses (Box 1).

Persistence and long-term immune stimulation in lymphoid tissue may also provide benefit to the host via prolonged stimulation and induction of durable immunity to reinfection [41,70]. Macaques infected with measles virus have persistent RNA in lymphocytes and myeloid cells for months after resolution of the acute rash disease. Pathologic examination of their lymph nodes shows a progressive increase in germinal centers with proliferating B cells accompanied by continued appearance of virus-specific peripheral follicular helper CD4^+^ T cells and antibody-secreting cells in circulation and affinity maturation of antiviral antibody [41]. This contrasts with the short-lived immunity induced by SARS-CoV-2 and many other respiratory viruses potentially due to a failure to establish the persistence of RNA in lymphoid tissue required for prolonged synthesis of viral antigens for immune stimulation [157–161].

## Concluding remarks

Clinical recovery, elimination of detectable infectious virus, and development of immunity after infection with RNA viruses that cause acute infections do not necessarily result in complete elimination of the viral RNA. Both virus and host mechanisms can prevent production of infectious virions while allowing persistence of viral RNA in previously infected cells. Viral mechanisms include mutations in genes coding for proteins required for assembly or replication and evasion of the adaptive immune response. Host mechanisms include the use of noncytolytic clearance mechanisms that allow infected cells to survive and cell type–specific activation of innate immune responses that suppress virus replication in infected cells. How RNA is protected from degradation is unclear, but occasional late transmission and continuing stimulation of adaptive immune responses indicate persistence of genomic and translatable viral RNA.

Our understanding of the long-term consequences related to disease and durable immunity and the mechanisms of persistence will benefit from further investigation and development of appropriate animal models. Future studies will be needed to identify the types and locations of cells harboring viral RNA and the metabolic state of these cells compared with uninfected cells. In addition, a better understanding of the state of the viral RNA, how it is protected from degradation, the relative amounts of full-length and DVG or fragmented RNA, and the contribution of continued RNA synthesis to persistence will help to solve this mystery and inform potential interventions. Identification of the role of RNA persistence in late disease could be advanced with longitudinal studies that evaluate treatments that suppress RNA replication and examine their effects on RNA persistence and long-term outcomes.

## Abbreviations

CNS: central nervous system
COVID: Coronavirus Disease
DVG: defective viral genome
HCV: hepatitis C virus
HIV: human immunodeficiency virus
IFN: interferon
LCMV: lymphocytic choriomeningitis virus
MHC: major histocompatibility complex
PASC: post-acute sequelae of COVID-19
RSV: respiratory syncytial virus
RT-PCR: reverse transcriptase polymerase chain reaction
SARS-CoV-2: Severe Acute Respiratory Syndrome Coronavirus 2
SSPE: subacute sclerosing panencephalitis
